# The power of e-recruitment and employer branding on Indonesian millennials’ intention to apply for a job

**DOI:** 10.3389/fpsyg.2022.1062525

**Published:** 2023-01-06

**Authors:** Cindy Natalia Wijaya, Martina Dwi Mustika, Sefa Bulut, Baidi Bukhori

**Affiliations:** ^1^Faculty of Psychology, Universitas Indonesia, Depok, Indonesia; ^2^Department of Counseling Psychology, Ibn Haldun University, Istanbul, Turkey; ^3^Faculty of Psychology and Health, Universitas Islam Negeri Walisongo Semarang, Semarang, Indonesia

**Keywords:** e-grocery, employer branding, employee value proposition, e-recruitment, intention, post-pandemic, experimental vignette method

## Abstract

Companies need reliable employees to support their business. As e-grocery businesses in Indonesia continue to grow during the pandemic, various strategies are required to attract millennials. This study aims to prove the influence of employer branding, e-recruitment, and post-pandemic employee value proposition (radical flexibility, deeper connection, personal growth, and holistic well-being) in encouraging millennials to apply for jobs at e-grocery companies after the pandemic. Few e-grocery companies in Indonesia use employer branding and e-recruitment; this study attempts to combine these two variables to see the effect of their interaction on influencing greater millennial intentions. Using the latest experimental method, which is the experimental vignette method, we conducted three studies with a total of 619 millennial participants, who were recruited using the convenience sampling technique. All participants received a set of job advertisements as a stimulus. The results showed that e-recruitment could not significantly predict the millennials’ intentions when applying for jobs in e-grocery companies. Companies in Indonesia may need to analyze millennials’ familiarity with e-recruitment platforms, especially on company websites. However, employer branding successfully predicted millennials’ intention to apply for a job in e-grocery companies, which was not affected by the length of their work experience. Employer branding serves as a means of building job seekers’ trust through personal promotions. With this trust, job seekers are more motivated to apply to the company. When e-recruitment and employer branding were analyzed simultaneously, there were significant interactive effects on millennials’ intentions. Employer branding acted as a socialization medium to introduce e-recruitment and vice versa. E-recruitment served as a form of branding that could shape the perceptions and experiences of millennial job seekers. Lastly, the employee value proposition significantly predicted millennials’ intentions, where holistic well-being was the most sought-after value. It can support employees’ well-being and encourage them to make valuable propositions that will make e-grocery companies excel in Indonesian labor market.

## 1. Introduction

The COVID-19 pandemic altered how Indonesians made their daily purchases. Various traditional lifestyles began to transform into technology based. Online shopping has also broadened to include grocery products such as vegetables and food ingredients. During the pandemic, 21% of Indonesians started to shift to using online grocery shopping services (DBS Group Research, 2020). This rate increased sevenfold during the COVID-19 pandemic, which also increased the development of businesses based on B2C, delivery apps, and digital and technology (Mercer, 2020; Riches, 2021). These businesses then should use an unique approach in recruiting talent. Highly qualified employees can help these businesses improve their consumer demand and satisfaction, not only with their products but also with their services. This is also a form of investment to increase the company’s long-term value.

The demand for new headcount increases by 14% (Michael Page Internasional Indonesia, 2021). However, around 90% of businesses struggle to recruit employees, including in Indonesia (CNBC Global CFO Council, 2021; Synthesis Academy, 2021). Technology and internet-based companies are experiencing great competition from larger enterprises such as baking, insurance, and business services companies’ sector (Michael Page Internasional Indonesia, 2021). The existence of a new perspective for Indonesian people toward work after the pandemic has also made companies struggle more to determine the most attractive recruitment strategy for them (Walters, 2021). Such as promoting well-being and work-life balance, liking flexible working arrangements, and having certain development programs. With this condition, technology-based companies in Indonesia must find the right strategy to seek out recruits, including e-grocery companies, which are starting to experience an increase in demand. One method to recruit highly qualified employees is using an effective recruitment system and employer branding (Bromberg and Charbonneau, 2020). One of the problems often faced by job seekers is the recruitment process that is too long and tedious, the lack of follow-up, and the lack of transparency of expectations from the company (Michael Page Internasional Indonesia, 2021). E-recruitment is considered one of the most effective methods in terms of cost, time, and accessibility in reaching many candidates (Sultana and Sultana, 2017).

In addition to the recruitment process, one of the problems that also occurs is the lack of information provided by the company to jobseekers regarding the vacancies opened. Through employer branding, companies can communicate who they are, their values and goals, and how they work with job seekers (Michael Page Internasional Indonesia, 2021). Employer branding, such as value proposition, can also be used to form company image, perception, reputation (Tikson et al., 2018), and attraction, which motivate job seekers to apply to that company (Kumari et al., 2020). However, doing branding is more challenging than imagined. According to Martic (2022), one of the biggest challenges in conducting employer branding is the need for more engagement of prospective employees with employer branding and marketing activities carried out by the company. Especially in remote conditions like this, it is increasingly difficult to get in touch personally with prospective employees and get them involved in the company’s branding efforts (Ranthee and Bhuntel, 2017). Therefore, we need a tool to reach out to the candidates directly, form a personal relationship with them, and increase their engagement. E-recruitment is one of the closest methods to job seekers and helps the employer branding process. Few e-grocery companies in Indonesia use employer branding and e-recruitment; this study attempts to combine these two variables to see the effect of their interaction on influencing greater millennial intentions. Both e-recruitment method and employer branding will be further investigated in the context of e-grocery businesses to predict Indonesian millennials’ intention to apply for a job.

However, it turns out that employer branding is perceived differently based on employees experience working in a similar field or their experience researching the company (Michael Page Internasional Indonesia, 2021). The more experience individuals have, the more different their perceptions of a company’s branding influence their intention to apply. The amount of experience is influenced by the length of time the jobseeker has been in the field. Therefore, the length of experience will be investigated further as a covariate of the influence of employer branding on the intention to apply.

As previously stated, a challenge in winning labor competition is the loss of benefits provided by technology sector companies, with larger sectors such as banking and insurance. The benefits provided by these two sectors tend to be greater than those of technology companies, making it more attractive for job seekers to apply. We need a benefit and company value that is more attractive and different than other companies. These benefits must also be communicated openly to jobseekers during the recruitment process through employer branding. One form of good communication in employer branding is to include a strong employee value proposition (EVP; Michael Page Internasional Indonesia, 2021). This employee value proposition must be structured in such a way based on the factors that most influence the job seeker’s perception of the company’s brand. The influencing factors are also different in each generation and era. With the pandemic conditions changing the lifestyles of job seekers, a post-pandemic employee value proposition has been prepared. This adjustment must be made, especially because Indonesian millennials tend to be frustrated when their companies do not provide what millennials expect (Deloitte Indonesia Perspectives, 2019). The post-pandemic value proposition is prepared in a more human-centric manner, so that it is more oriented toward treating employees as individuals, not just workers (Gartner, 2021). With the characteristics of Indonesian society that prioritizes society and individual welfare, the post-pandemic employee value proposition can be a novelty in itself to be applied to Indonesian millennial workers (Cultural Atlas Editors, 2016). This study will be further investigated the effect of post-pandemic employee value proposition in e-grocery businesses to predict Indonesian millennials’ intention to apply for a job.

The millennial generation, born between 1981 and 1996 (Badan Pusat Statistik, 2021), dominates the job market at around 33.75% and is the largest portion of Indonesian workers compared to other generations (Badan Pusat Statistik, 2017). This generation use technologies and the Internet in their daily lives and find it easy to adapt to new technological advancements (Vogels, 2019; Whatton, 2019; Indeed Editorial Team, 2021). Their Internet familiarity helps them easily find jobs from various job portals, including *via* an information and technology-based recruitment method. They easily obtain job vacancy information, apply to a company, and follow its e-recruitment processes. These fast e-recruitment processes then are more likely to attract millennials and increase their motivation to apply (Whatton, 2019).

Intention to apply for a job is the process of a candidate’s interest in joining a company. It involves collecting information on the company, studying the vacant position, and sending job application documents to the desired company (Barber, 1998). The Theory of Planned Behavior suggests that intention is formed by two factors: an individual’s attitude toward the behavior that will be carried out, and society’s subjective norm in assessing whether that behavior is good (Ajzen, 1985). In applying for a job, a candidate’s attitude may be formed by how they evaluate the information given by the prospective company on the job advertisement from both individual and social perspectives (Barber, 1998; Erlinda and Safitri, 2020). The more positive the individual’s evaluation of the information, the greater their intention will be. Therefore, a company’s information has a vital role in promoting a candidate’s job application intention, and this information can be found in e-recruitment and employer branding.

E-recruitment is a series of coordinated and systematic recruitment activities using technologies (Abia and Brown, 2020). Using this method, job seekers will obtain information through online media (such as social media, employment platforms, and company websites). They then submit their applications online (*via* email or direct website). Finally, technology will help the recruiter to do candidates’ screening, selection, and contact them quickly and automatically (Sills, 2014; Rimadias and Pratiwi, 2017). Conventional recruitment method, on the other hand, requires jobseekers to obtain vacancies information through printed media (newspapers, brochures, billboards) and send their application to the company’s address. The recruiter then must screen and select their prospective candidates manually (Sivabalan et al., 2014). Different from conventional recruitment which, e-recruitment may be conducted through two types of platforms: the company’s official website and a commercial jobs board such as LinkedIn, JobStreet, Facebook, or Indeed (Kaur, 2015). In e-recruitment, companies can undergo several things at once: (1) displaying updated and detailed information on the vacant positions; (2) helping candidate employees observe the application status that is delivered periodically through the real-time information feature (Muthuveloo et al., 2017); (3) reaching a wide scope of candidates as Internet access is not limited to a certain space and time, especially when using certain job portals; (4) organizing certain competence tests and comprehensively evaluating candidates (not only based on the resume they sent); and (5) helping companies and candidate employees connect and interact. The last advantage will help them develop broader career opportunities (Jayachandran and Devi, 2016). These advantages could make e-recruitment an effective, easy, and quick recruitment method. Previous research showed that e-recruitment positively influenced candidates’ intentions when applying for jobs (Luyanage and Galhena, 2014; Muthuveloo et al., 2017; Rimadias and Pratiwi, 2017; Ekanayaka and Gamage, 2019). The availability of more regularly monitored factual information (the use of which is fast, easy, and cost-effective) and the wide range of job information make e-recruitment a unique attraction to increase the intention of job seekers to apply (Muthuveloo et al., 2017; Sabha, 2018).

Employer Branding is the process of developing an identifiable and unique company identity that differentiates it from others (Backhaus and Tikoo, 2004). There are two types of employer branding: internal and external. Internal employer branding is directed to employees who have joined the company. The company tries to increase a sense of belonging to solidify an employee’s commitment to that company (Punjaisri and Wilson, 2011). The positive attitude shown by current employees toward the company will also become the company’s point of interest for the candidate, which later forms external employer branding (Chhabra and Sharma, 2014). External employer branding represents the identity, condition, job description (Wright, 2021), and the perspectives of working employees (Nagpal and Nagpal, 2019) through advertisement and marketing strategies (Einck, 2018). It can be found on websites, social media, corporate social responsibilities, seminars, and advertisement agencies, such as television, radio, or sponsorship cooperation (Morya and Yadav, 2017). External employer branding is designed to attract candidates and should be memorable, meaningful, protectable, and adaptive to the current era (Chhabra and Sharma, 2014; Weerawardane and Weerasinghe, 2018a,b). In Indonesia, 45% of job seekers tend to do prior research before applying to the company (Michael Page Internasional Indonesia, 2021), and all this attractively designed information is one of the considerations for candidates to adjust their needs with what is offered by the company.

Length of experience is the amount of time a person has spent working in a particular industrial sector (Metadata Online Registry, 2007). The length of experience and experience that a person goes through can affect how a person views life phases, personal conditions, and the things that make him interesting (Emmet et al., 2021). Thus, a company must be able to design a flexible and personal experience in a recruitment and branding process so that every job seeker is interested in considering the company based on their interests and needs. The suitability of experience with the company’s branding will help increase the intention of job seekers to apply for the company.

One component that is communicated in employer branding and that attracts employees’ intentions is the employee value proposition. The employee value proposition is a series of benefits or values that employees will obtain when working at a certain company (Heger, 2007). During the pandemic, employees experienced different conditions and needs. Gartner (2021) defined five post-pandemic employee value propositions that employees prefer during a pandemic. Adjusting the value propositions that best suit job seekers’ current conditions can increase job seekers’ intentions to apply. Each of these types has different benefits and effects; however, considering the within-subject experimental design in this study, the researchers only chose four types to analyze. These four types were selected based on the most in-demand benefits by employees from previous studies (Sivertzen et al., 2013; Jain and Bhatt, 2015; Amelia and Nasution, 2016; Widyaningsih, 2016; Abid and Barech, 2017; Gilani and Cunningham, 2017; Kusuma and Prasetya, 2017; Kumari et al., 2020; Shah et al., 2020; Willis, 2022).

The first type is deeper connection. During a pandemic, employees need a personalized relationship that establishes certain boundaries. To ensure that they remain responsible for their duties and commit fully to working, companies must provide more comprehensive benefits, including to the workers’ families. Thus, trust is created between employees and the company (Gartner, 2021). Additionally, the pandemic shifted the priorities of employees to valuing their familial and communal relationships more, so providing opportunities that can strengthen these relationships will be more meaningful. Companies that allow time for deeper connections will help employees feel understood and valued.

The second is radical flexibility. During a pandemic, many companies offer flexibility regarding time and place of work. This condition is intended to increase employees’ motivation and work–life balance (Jain and Bhatt, 2015; Amelia and Nasution, 2016); however, to improve the performance of employees as a team, team-based flexibility is needed. Radical flexibility will encourage the employees’ freedom by providing flexibility in various work aspects, such as work division, teamwork methods, and the place and time of work.

The third is personal growth. Fifty percent of employees in the workplace feel it is important to have the opportunity to develop themselves while working (Gartner, 2021). This self-improvement during a pandemic can help employees maintain performance by increasing their skills, even though they are working from home. However, it is not only limited to a professional context; psychosocial and interpersonal self-development is also considered important to improving performance and maintaining mental health (Bains, 2021). This employee value proposition ensures that each employee is respected by helping them grow as social beings instead of merely professional beings.

The fourth is holistic well-being. During a pandemic, one of the most affected aspects is employees’ mental health which is impacted regardless of age, position, and extent of work. Mental health is impacted especially when experiencing sudden changes in working conditions that limit community mobility (such as working from home). Sometimes this condition is exacerbated by uncontrolled working hours, which can lead to stress and burnout (Platts et al., 2022). This condition makes the company strive to provide various benefits to support employees’ mental health. Even the benefits offered also support physical, financial, and emotional aspects (Gartner, 2021). But in reality, only a few takes advantage of these benefits. For this reason, a holistic well-being employee value proposition exists that will care for the employees by providing facilities that support physical, social, psychological, and spiritual welfare. They will also ensure that their employees use those facilities. This study aims to prove the influence of employer branding, e-recruitment, and post-pandemic employee value proposition (radical flexibility, deeper connection, personal growth, and holistic well-being) in encouraging millennials to apply for jobs at e-grocery companies after the pandemic.

Currently, there are approximately 140 e-grocery companies in Indonesia, including HappyFresh, Segari, Sayurbox, TaniHub, and Ula (Tracxn, 2022). However, not much e-grocery companies in Indonesia use the e-recruitment method and maximize all e-recruitment features (for example, only using online media to share job vacancy information, but still asking candidates to send their applications directly to the company). As a company that sells their products online, it can be said that the e-grocery company strengthens the online ecosystem’s image in people’s daily lives (Aull et al., 2021). With the online ecosystem is becoming more ubiquitous, it makes the use of e-recruitment more relevant to e-grocery companies. As such, e-grocery companies in Indonesia should be quite familiar with this method.

In addition, as a generation that has grown up with technology, it is easy for millennials to adapt to all things related to the Internet (Santiago, 2019) and have specific expectations regarding the use of technology in the workplace (York, 2017). They expect a fast process and instant communication, which can be done anywhere and at any time (Mery, 2019). Thus, when the recruitment process switches to e-recruitment that can provide convenience, effectiveness, and real-time information, it becomes the main attraction for millennials to apply.

*Hypothesis* 1: E-recruitment method can predict millennials’ intention when applying for a job at an e-grocery company.

Employer branding may also be a source for obtaining information on a company. Previous research showed that employer branding influenced candidates to apply for certain jobs (Amelia and Nasution, 2016; Ruchika and Prasad, 2017; Purusottama and Ardianto, 2019; Kucherov and Zhiltsova, 2021). It increases a company’s reputation and familiarity with the candidates, and thus plays a deciding factor in the application (Saini et al., 2015). A powerful employer branding will make a company superior to others, especially in attracting the market’s interest (Weerawardane and Weerasinghe, 2018a,b). Millennials tend to identify modern and different employer branding on companies’ social media as more meaningful (Völker, 2018; Kucherov and Zhiltsova, 2021). Therefore, the branding carried out by a company will make millennials more familiar with that company and will motivate them to apply to that company.

*Hypothesis* 2: Employer branding predicts millennials’ intentions when applying for jobs at an e-grocery company.

Both e-recruitment and employer branding can become instruments to recruit diverse and qualified candidates (Bromberg and Charbonneau, 2020). Apart from having their own influences, e-recruitment and employer branding also interact, resulting in a greater influence on the intention of millennial employees. E-recruitment may also become a form of company branding (Zahrudini and Afrianty, 2020). Previous research has shown that e-recruitment and employer branding can influence each other (Weerawardane and Weerasinghe, 2018a,b; Zahrudini and Afrianty, 2020). Companies will have a good reputation and be deemed effective in undergoing recruitment, thus encouraging job seekers to send in their job applications. The use of technology from e-recruitment can also be a separate tool to attract and retain the interest of job seekers. In addition, it can be a supporter of employer branding activities (Lievens and Slaughter, 2016). The use of technology from e-recruitment can also be a separate tool to attract and retain the interest of job seekers. In addition, it can be a supporter of employer branding activities. Meanwhile, the presence of information on a company (shown through employer branding activities) will ease candidate employees into understanding the company and become one of the tools that make it easier for job seekers to do research before applying since researching before applying is one of the tendencies carried out by millennial jobseekers (Michael Page Internasional Indonesia, 2021).

Seeing the important roles of these two variables, this research also aims to see whether e-recruitment and employer branding have interactive effects that influence the intention of millennial employees. This is because previous research showed no interactive effects in influencing employee intention when employer branding was tested with company reputation (Sivertzen et al., 2013). These two variables are in a series of recruitment processes. Thus, the two may be shown simultaneously, and the interaction in influencing the intention of candidate employees can be analyzed (Jobvite, 2015).

The existence of a good company reputation, high interest, and the effectiveness of accessing specific information will affect millennial intentions in applying. Despite the presence of attractive and competitive employer branding, how an individual’s perspective depends on their work experience. Individuals with longer work experiences will be more reflective and critical in evaluating the employer branding signals (Wilden et al., 2010; Modi and Patel, 2012; Zahrudini and Afrianty, 2020; Kismono and Rahayu, 2021; Miradewi and Fitriani, 2021). We then suggest these two hypotheses:

*Hypothesis* 3: E-recruitment methods and employer branding can predict a millennial’s intention when applying for a job at an e-grocery company.

*Hypothesis* 4: Employer branding still can predict a millennial’s intention when applying for a job at an e-grocery company when the length of work experience is controlled.

In employer branding, companies may also include employees’ value proposition, which is an effective instrument in developing reputation. Previous research showed that job seekers prefer companies that emphasize social values (Kumari et al., 2020) and the presence of support from colleagues and leaders (Kusuma and Prasetya, 2017). A friendly work environment can build a perception of support and make employees comfortable enough to perform optimally (Kusuma and Prasetya, 2017). In addition, having an open relationship with superiors can also reduce workers’ stress and create a conducive working environment (Kusuma and Prasetya, 2017; Kumari et al., 2020). Previous research also reveals that employees preferred to have flexible working hours (Kusuma and Prasetya, 2017). The existence of a flexible working arrangement can increase the work engagement level, which in turn can improve team performance (Shah et al., 2020). In addition, flexible working arrangements can also increase job satisfaction and quality of life among employees (Abid and Barech, 2017). This will affect the work–life balance that is expected by employees (Jain and Bhatt, 2015; Amelia and Nasution, 2016). Studies have found that the existence of self-development and the development of others is a distinct workplace preference (Sivertzen et al., 2013; Jain and Bhatt, 2015). The existence of personal development in the workplace can increase employee motivation, improve performance by increasing skills, and improve team performance through innovation, growth, and creativity (Willis, 2022). Employees expect a safe work environment (Amelia and Nasution, 2016) and competitive wages and other compensations (e.g., bonuses, incentives, insurance, and retirement funds; Widyaningsih, 2016; Gilani and Cunningham, 2017).

Millennials prefer company management, culture, and benefits (PWC, 2011). Employee value proposition helps call attention to the needs of employees (Goswami, 2015). Thus, employees’ intention to apply for a job will differ depending on their needs and those provided by the companies. Millennials also have quicker attention spans, communication styles, and ways of life that differ from other generations. They may expect the provision of self-development programs or career changes (Gallup, 2016) and have the desire to keep on learning while working (Whatton, 2019). Therefore, 87% of millennials also regarded growth in workplaces and careers as crucial in their job. They always want to be involved and have roles in their work (Gallup, 2016). We then propose the following hypotheses:

*Hypothesis* 5a: There are significant differences between each employee value proposition type in predicting the intention of millennial employees applying for jobs in e-grocery companies.

*Hypothesis* 5b: Personal growth is the most influential employee value proposition in predicting the intention of millennial employees applying for a job at an e-grocery company.

This research focuses on seeing the intention of millennial employees when applying for a job at an Indonesia e-grocery company that several variables can predict. We conduct three studies using the experimental vignette method within-subject design to achieve this. The experimental vignette method is a method that exposes participants to a structured and realistic scenario to test intention, behavior, and attitude. This method allows the researcher to manipulate and control independent variables, thus increasing internal and external validities (Atzmüller and Steiner, 2010; Aguinis and Bradley, 2014). This method will also allow to provide further causal support in Indonesian context. The first study aims to see the influence of e-recruitment, employer branding, and the interaction between e-recruitment and employer branding in predicting the intention of millennial employees (Hypothesis 1, 2, and 3). The second study aims to see the role of work experience length in moderating the influence of employer branding on millennial employees’ intentions (Hypothesis 4). Lastly, the third study aims to observe the influence of the post-pandemic employee value propositions in predicting the intention of millennial employees (Hypothesis 5a and 5b).

This research has obtained an Ethical Clearance from the Committee of Ethics, Faculty of Psychology (No. 155/FPsi.Komite Etik/PDP.04.00/2021). The stimuli of this research were in the form of online job vacancy advertisements, which were designed according to the manipulated independent variables of each study, to measure the intention of candidate employees to join that company. The manipulated online job vacancy advertisements and surveys function to measure the independent variables. We used online job vacancy advertisements as they can reach more suitable candidates, they can be easily accessible from various gadgets, the information they contain is easily seen and understood, and they allow for more dynamic creation, thus allowing various manipulations (Phillpott, 2021). All stimuli in this research were arranged based on focus group discussions (FGDs) with eight millennials from various backgrounds to investigate their attitudes, feelings, beliefs, and reactions. The FGD method is also suitable for use concerning attitudes, behaviors, and motivations (Copley Focus Centers, 2012).

The Intention Toward Company (*α* = 0.82) instrument was used to measure the intention to apply for the job (Highhouse et al., 2003), which consisted of five items with the 1–5 Likert scale (strongly disagree to strongly agree). This instrument was adapted to the Indonesian language using the procedures from Sousa and Rojjanasrirat (2011). Survey Monkey online platforms were used to display the stimuli and to allow participants to fill the instrument. Participants accessed a link shared through social media such as LINE, Instagram, Facebook, LinkedIn, and WhatsApp. Counterbalancing was also carried out by asking participants to choose numbers from 1 to 10 that they liked, which will direct them to questionnaires with different orders for each number.

Participants in this research were millennial employees born between 1981 and 1996 (Badan Pusat Statistik, 2021). The participants were chosen using the convenience sampling technique, with a target of 200 participants for each study (Brysbaert, 2019). Every participant who followed the research until the end had the opportunity to join a lucky draw with the prize of electronic money. From each study, 10 participants were randomly chosen to win the electronic money lucky draw in the amount of Rp50.000,00 each.

Generally, when accessing the questionnaire link, participants will directly see an informed consent that will explain shortly (1) the aim of the research that will be carried out, (2) a short description of the studied topic, (3) the freedom of the participants to participate in this research and to end it at any time, (4) what they will face in the questionnaires, (5) the freedom of the participants to answer each item according to their conditions, (6) data confidentiality, and (7) the compensation they will receive, including their freedom to participate in the lucky draw or not. After reading the informed consent and after stating that they agreed to participate in this research, the participants filled in information on their demographic data, including the year they were born, their gender, their city of domicile, and length of work experience. Then, they were directed to a series of stimuli, manipulation check questions, and measurement instruments consecutively. At the end of the study, the participants were given a debriefing and a separate link for those who wished to join the lucky draw.

## 2. Study 1

### 2.1. Method

In this study, 210 millennials (*M*_age_ = 31.82, SD = 4.314, male = 40.5%, female = 57.1%) participated in the 2×2 study with the variation of stimuli. Participants received seven-page questionnaires consisting of informed consent, a place to fill demographic data, four pages of stimuli, a debriefing page, a manipulation check, and an Intention Toward Company scale. The manipulation check in this study consisted of two questions in the form of multiple-choice: “Does the company explain how employees usually work in that company?” and “How do you apply to that company?” The variation of the e-recruitment variable consisted of e-recruitment and conventional recruitment. Then, the employer branding variable consisted of these variations: with employer branding and without employer branding. These two variables were combined into four stimuli in the form of job vacancy posters with these variations: (A) with employer branding with an e-recruitment method, (B) with employer branding with a conventional recruitment method, (C) without employer branding with an e-recruitment method, and (D) without employer branding with a conventional recruitment method. Generally, the stimuli contained the company’s logo and tagline, a short description of the company, and how to apply to that e-grocery company.

**Figure 1:**
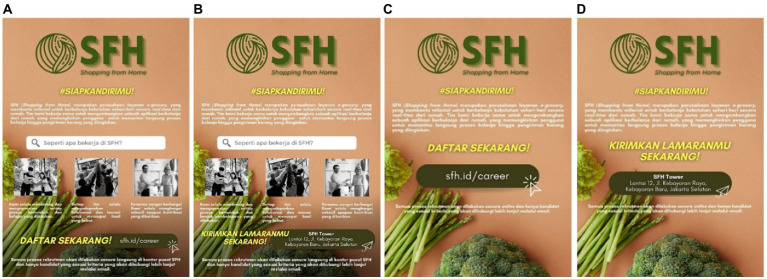
The stimuli for study 1. Variations: **(A)** with employer branding with an e-recruitment method, **(B)** with employer branding with a conventional recruitment method, **(C)** without employer branding with an e-recruitment method, and **(D)** without employer branding with a conventional recruitment method. All elements used in this image are created from Canva.com with subscription on 25 October 2021. It is copyrighted by Cindy Natalia Wijaya and may not reproduced without permission.

The difference in the stimuli lies in whether there is a job description and the method of applying to that company. Figures 1A,B contained a short job description representing various employer branding. The job description was delivered through three main values of the companies that were implemented in how the employee’s work. There were also photos of employees who had worked in that company. Then, in Figures 1C,D because it was as if the company did not have employer branding, there was no further information on the company. There was only a short description of that company’s industrial sector. Then, in Figures 1A,C, it was as if the company used the e-recruitment method to recruit new employees. Thus, there was a link to the company’s website where applicants may send their job applications. This website link also showed that the recruitment process was carried out online. Then, in Figures 1B,D, the company used the conventional recruitment method, thus it contained the company’s address to which applicants may send their job applications.

### 2.2. Data analysis

The analysis was carried out using the factorial analysis of variance (ANOVA) repeated measure technique. This technique was chosen because two independent variables were manipulated, and each participant experienced several measurements from different manipulated conditions. In the end, they had several measurement scores. This analysis technique assumed that the difference in an individual’s score variation was partly due to the different manipulations employed. The end results of this analysis were the main effect that explained the influence of each independent variable on the dependent variable and the interaction effect that explained the influence of the interaction between the two independent variables on the dependent variable (Field, 2013).

### 2.3. Results

In undergoing the analysis, Mauchly’s Test of Sphericity for the recruitment method and employer branding cannot be employed as these two variables only have two variations. These two variations showed that the Sphericity Estimate was 1 (non-violated), the chi-square was 0, and there were no degrees of freedom; thus, the value of *p* cannot be calculated. Therefore, the degree of freedom used is Sphericity Assumed.

Based on Table 1, it can be concluded that there were no significant main effects from the e-recruitment method in predicting millennials’ intentions when applying for a job at an e-grocery company, *F*(1, 209) = 2.004, *p* = 0.158. The intention of millennials when applying to the job was not very different from when the company used e-recruitment (*M* = 17.533, SE = 0.265, 95% CI = [17.911, 18.056]) and when it used the conventional recruitment method (*M* = 17.329, SE = 0.249, 95% CI = [16.838, 18.056]). Thus, hypothesis 1 was not supported. But there was a significant main effect from the company’s use of employer branding at the e-grocery in predicting millennials’ intention when applying for a job, *F*(1, 209) = 25.714, *p* = 0.000. The intention of millennials in applying for the job was significantly higher when the company provided employer branding (*M* = 17.938, SE = 0.267, 95% CI = [17.412, 18.464]) compared to without employer branding (*M* = 16.924, SE = 0.265, 95% CI = [16.401, 17.447]). This shows that hypothesis 2 is supported.

**Table 1:** Tests of within-subjects effects for study 1.

Source	Type III sum of squares	df	Mean square	*F*	Sig.	Partial eta squared
Metode_Rekrutmen (Recruitment method)	8.805	1	8.805	2.004	0.158	0.009
Error	918.195	209	4.393			
Branding	216.043	1	216.043	25.714	0.000	0.110
Error	1755.957	209	8.402			
Metode_Rekrutmen (Recruitment method) * Branding	29.719	1	29.719	8.127	0.005	0.037
Error	764.281	209	3.657			

**Figure 2:**
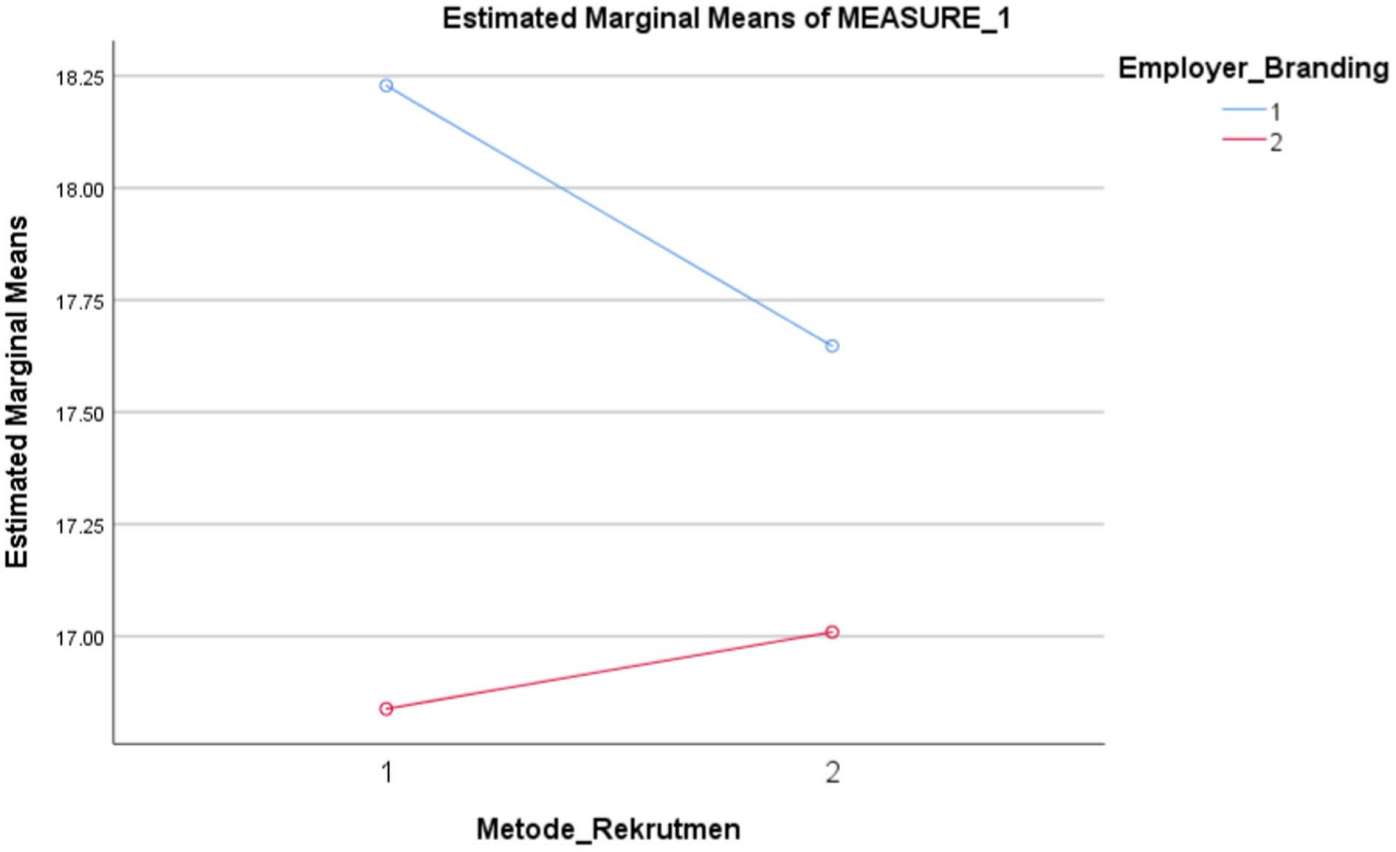
Descriptive statistics. The vertical axis shows the estimated marginal means of the dependent variable, namely the intention to apply. The line shows the level of intention to apply for jobs among millennials. While horizontal axis shows the first variable, namely method of recruitment. The numbers on the horizontal axis are the code for the variations, one for e-recruitment and two for conventional recruitment. The color of the line shows the variation of the second variable, namely employer branding. Blue for with employer branding and red for without employer branding. The intersection of the lines shows the interaction effect between the two variables. All elements used in this image are created from Canva.com with subscription on 25 October 2021. It is copyrighted by Cindy Natalia Wijaya and may not reproduced without permission.

There was a significant interaction effect between recruitment methods and employer branding in predicting millennials’ intention when applying for a job at the e-grocery company, *F*(1, 209) = 8.127, *p* = 0.005. Based on Figure 2, it can be explained that in general, the intention of millennials toward companies with employer branding was higher than companies without employer branding. The intention of millennials was higher when companies used the employer branding and the e-recruitment method (*M* = 18.23, SD = 4.138, SE = 0.286, 95% CI = [17.666, 18.792]) compared to if they only used the conventional method (*M* = 17.65, SD = 4.140, SE = 0.286, 95% CI = [18.084, 18.211]). When companies do not have employer branding, the millennials’ intention will generally decrease. But the intention increased when it used the conventional recruitment method (*M* = 17.01, SD = 3.909, SE = 0.270, 95% CI = [16.478, 17.541]) compared to when they used the e-recruitment method (*M* = 16.84, SD = 4.244, SE = 0.293, 95% CI = [16.261, 17.415]). In conclusion, hypothesis 3 was supported.

### 2.4. Discussion

For Indonesian millennials, the use of the e-recruitment method does not predict their intentions to apply for a job at an e-grocery company, especially when e-recruitment is done on the company website. Even though millennials are categorized as a generation with a high Internet consumption rate, 91.30% of Indonesian millennials only use the Internet for socializing *via* chatting features. Only 8.9% of millennials use the Internet to look for job opportunities (Alvara Research Center & IDN Media, 2020). This result brings a new explanation why Indonesian millennials are not yet familiar with e-recruitment (especially on company websites) as a method to apply for a job. It was also found that Indonesian job seekers are usually more familiar with participating in e-recruitment through certain job platforms such as JobStreet (Rimadias and Pratiwi, 2017). The use of e-recruitment and company websites as e-recruitment media must be considered for socialization because it will help e-grocery companies to increase their attractiveness through the communication of their work culture and position themselves as the company of choice (Fountain, 2022).

The use of employer branding in the e-grocery company can predict the intention of Indonesian millennials when applying for jobs. This is in line with many previous studies (Amelia and Nasution, 2016; Ruchika and Prasad, 2017; Purusottama and Ardianto, 2019; Kucherov and Zhiltsova, 2021). Millennials behave as consumers of the workplace, weighing their options and continually looking for roles and organizations that enable their best performance (Gallup, 2016). According to Fairhurst (2022), one way for an e-grocery company to stand out in the market is by building a special connection with customers. This connection can be achieved by having a personalized promotion that establishes trust and can be used by companies to succeed in the market. This concept also occurs in the labor market. The result brings a new explanation that an e-grocery company needs to build a connection by conducting personalized promotions to win in the labor market. Employer branding contains a variety of personalized information and can build job seekers’ trust so that a relationship is created that can encourage their intention to apply. Personalized promotion is reflected when the company tries to adapt these three things to millennials’ personality, lifestyle, and social values (Putritamara et al., 2022). This form of personalization can build subjective perceptions of millennials and determine whether they are suited to the organization. The more suitable the characteristics of the company are to themselves, the higher the intention to apply to that company, especially when companies are more inclined to an emotional approach. When companies use the emotional approach to promote their brands, millennials tend to empathize and try to adapt to the characteristics and values required by the company (Putritamara et al., 2022).

In addition, in employer branding, there is credible information on the organization’s characteristics, such as company values, goals, and missions (Kaur and Dubey, 2014), which can build a sense of trust among job seekers. Moreover, employer branding is usually displayed on company websites or job fairs. These two media are deemed as the most credible in providing information, thus they can encourage the intention of millennials to apply (Kaur and Dubey, 2014). Apart from the information on the company’s values, goals, and missions, subjective perception may also be obtained from the review of employees that appeared in employer branding. Employee reviews are deemed realistic information that can increase an organization’s attractiveness, thus increasing the intention of millennials to apply for a job (Kaur and Dubey, 2014).

The simultaneous usage of e-recruitment and employer branding in the e-grocery company can predict millennials’ intention to apply for a job. The use of employer branding can help companies communicate the recruitment methods they use, so the candidates will be more familiar with the platform used and the effective process of e-recruitment and encourage them to apply for a job at that company. The prepared value from the recruitment process will increase as they are associated simultaneously with the benefits offered by the company (Sabha, 2018). Contrarily, without socialization and branding toward the recruitment method chosen, the candidates will tend to choose the conventional recruitment method as it is deemed more familiar. Then, a recruitment method may also be used as the company’s branding instrument. If the recruitment method is simple and easy to navigate, companies will ensure candidates have a pleasant experience and a positive perception of the company brand. If the recruitment method is difficult and not properly organized, the company brand will be negatively perceived and thus decrease millennials’ intention to apply (Emerhub, 2017). The reciprocal relationship of these two variables brings new findings that e-recruitment and employer branding must be applied together in e-grocery companies to increase the intentions of millennial workers.

## 3. Study 2

### 3.1. Method

Study 2 measured the influence of the length of work experience as the covariate on the relationship between employer branding and millennial job seekers’ intention. In this study, 200 millennials (*M*_age_ = 31.62, SD = 4.231, male = 44%, female = 55.5%) were faced with a 1×2 study with the stimuli that were the same as Study 1, but only two stimuli were used: one with employer branding and another without. It’s intended because study 2 wants to investigate further the results of study 1, which found that employer branding affects millennial job seeker intentions. The stimuli contained the company’s logo and tagline, a short description of the company, and how to apply for a job at that e-grocery company.

**Figure 3:**
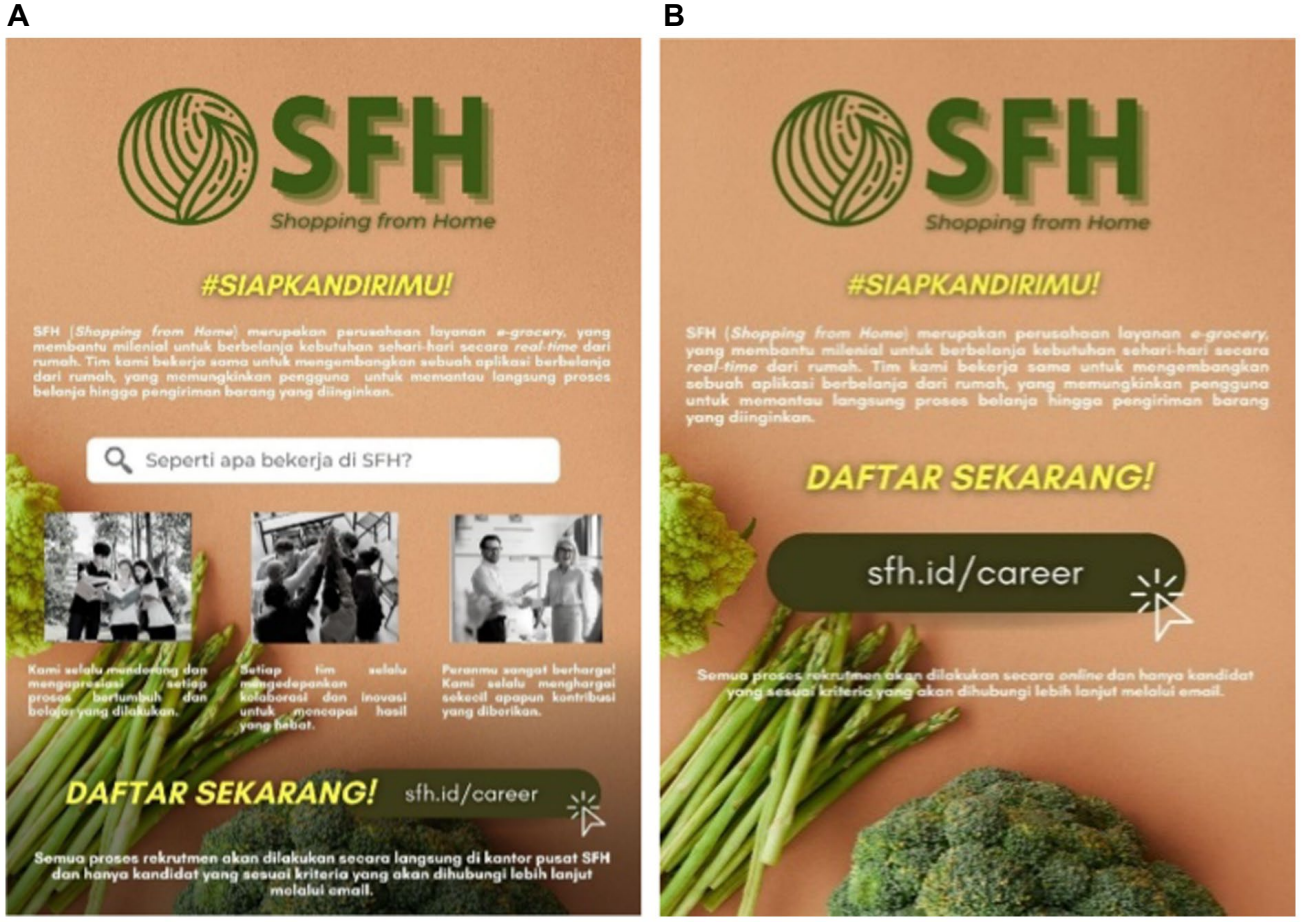
The stimuli for study 2. Variations: **(A)** with employer branding and **(B)** without employer branding. All elements used in this image are created from Canva.com with subscription on 25 October 2021. It is copyrighted by Cindy Natalia Wijaya and may not reproduced without permission.

Employer branding is shown in the form of a short description of the three main points a company brought and implemented in how the employees worked. Figure 3A showed that the company had employer branding, while there was no employer branding in Figure 3B. For Study 2, the manipulation check consisted of two questions with multiple choices, “The advertisements above are job vacancies in a company…” and “Does the company explain how employees usually work in that company?”

### 3.2. Data analysis

This study employed the analysis of covariance (ANCOVA) technique. This technique was chosen because the covariate in the length of work experience was also analyzed to see its influence on the dependent variables. The covariate was a continuous variable that was not part of the experiment’s manipulation but also influenced the dependent variables. This analysis tested the different means between treatments that have been adapted with the covariate’s influence (Field, 2013).

### 3.3. Results

In line with Study 1, Study 2 showed that the use of employer branding at e-grocery companies significantly predicted millennials’ intention to apply for a job. With these research results, testing was further carried out on new samples to see the influence of the length of work experience as a covariate variable. Table 2 describes the results of the calculation. Mauchly’s Test of Sphericity for employer branding could not be employed in the analysis as the independent variables only had two variations. These two variations showed that the Sphericity Estimate was 1 (non-violated), the chi-square was 0, and there were no degrees of freedom; thus, the value of *p* cannot be measured. The degree of freedom used is Sphericity Assumed.

**Table 2:** Tests of within-subjects effects.

Source	Type III sum of squares	df	Mean square	*F*	Sig.	Partial eta squared
Employer_branding	36.382	1	36.382	7.704	0.006	37
Employer_branding * Lama_Pengalaman (Length of experience)	388	1	388	82	0.775	0
Error	935.110	198	4.723			

**Figure 4:**
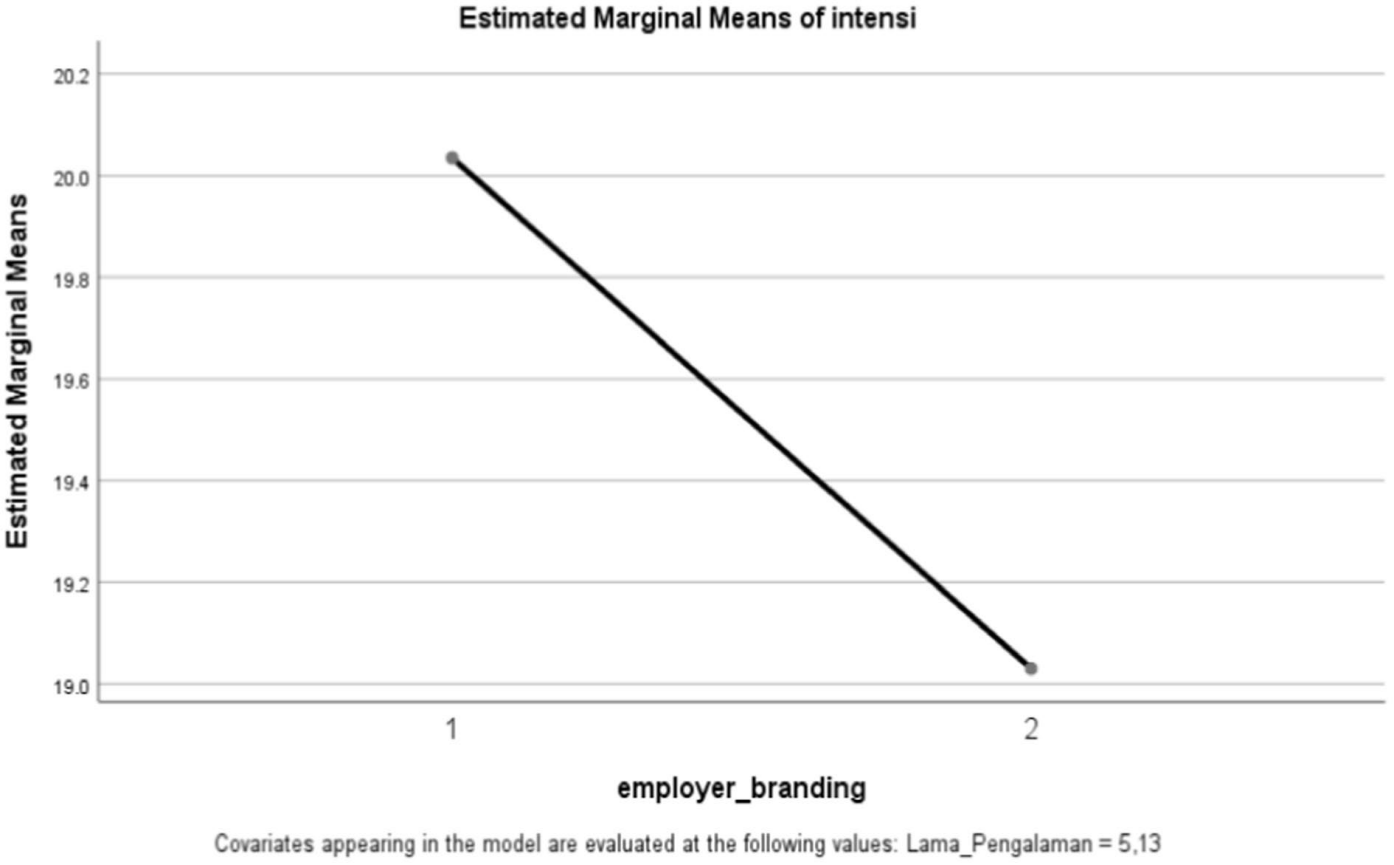
Descriptive statistics. The vertical axis shows the estimated marginal means of the dependent variable, namely, the intention to apply. The line shows the level of intent to apply for jobs among millennials. The horizontal axis shows the first variable—employer branding. All elements used in this image are created from Canva.com with subscription on 25 October 2021. It is copyrighted by Cindy Natalia Wijaya and may not reproduced without permission.

Based on Table 2 and Figure 4, it can be shown that there was a significant main effect from the company’s use of employer branding at the e-grocery in predicting millennials’ intention to apply for a job, as in Study 1, *F*(1, 198) = 7.704, *p* = 0.006, where millennial intentions are higher when the company has employer branding (*M* = 20.03, SD = 3.251, SE = 0.230, 95% CI = [19.582, 20,488]) compared to when the company does not have employer branding (*M* = 19.03, SD = 3.821, SE = 0.270, 95% CI = [18.498, 19.562]. But the results also showed that there was no significant difference in millennial intention when the covariate in the form of the length of the work experience was controlled, *F*(1, 198) = 0.082, *p* = 0.775. It can then be concluded that the length of work experience does not adjust the relationship between employer branding and millennials’ intention to apply for a job. Thus, hypothesis 4 was rejected.

### 3.4. Discussion

Although millennials have varying lengths of work experience, how they perceive a company tends to be the same as a generation. Regardless of whether a millennial has a lot of experience, they are more influenced by their latest job experience in choosing new jobs for the future (Florentine, 2017). This experience serves as a means for millennials to determine whether the job they are applying for is a job they want to pursue in the future and make them more aware of the requirements (Buzzeo and Cifci, 2017). In addition, the latest work experience can be used to compare their previous jobs and those they want to apply for and see how much control they have over their future careers (Bacon, 2015). Thus, the presence or absence of experience has more influence on how millennials perceive the company than the length of experience they have. This finding brings novelty that e-grocery companies must consider millennials’ quality of experience (than their length) and adjust the form/content of their employer branding according to the targeted job seekers.

## 4. Study 3

### 4.1. Method

In this study, 209 millennials (*M*_age_ = 30.28, SD = 4.840, male = 37.3%, female = 61.7%) were faced with a 1×4 study with one manipulated variable: employee value proposition. This variable was manipulated with these variations: radical flexibility, deeper connection, personal growth, and holistic well-being. Generally, the stimuli in this study showed photos of employees and their testimonies after working in that company for some time. These testimonies were different based on the benefits they obtained. These benefits were adapted to the value propositions of the company. Below is some information on the company and how to apply to that company.

**Figure 5:**
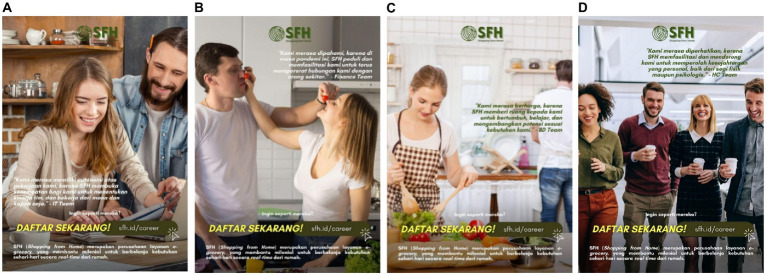
The stimuli for study 3. Variations: **(A)** radical flexibility, **(B)** deeper connection, **(C)** personal growth, and **(D)** holistic well-being. All elements used in this image are created from Canva.com with subscription on 25 October 2021. It is copyrighted by Cindy Natalia Wijaya and may not reproduced without permission.

In Figure 5A, the radical flexibility value shown from the employees’ expression is experiencing autonomy in managing their work and how they work. Then, in Figure 5B, the deeper connection value was shown from the employees’ expressions that they were facilitated to strengthen their connection with their surroundings during the pandemic. Then, in Figure 5C, the personal growth value was shown from the employees’ expressions who felt facilitated in learning new things, even outside of their jobs. Lastly, in Figure 5D, the holistic well-being value was shown from the employees’ expressions who felt facilitated and encouraged to obtain welfare according to their needs. These values were also implicitly presented in the theme of the employees’ photos, who gave testimonies for this advertisement. For Study 3, the manipulation check consisted of the multiple-choice question, “What do the employees obtain when working for that company?”

### 4.2. Data analysis

This study employed the one-way ANOVA repeated measure technique of analysis, which is different from study 1 and 2. This technique was chosen because there was only one manipulated independent variable with more than two variants, and each participant experienced several measurements from these differently manipulated conditions. In the end, participants had several measurement scores. This technique of analysis assumed that the difference in the variations of an individual’s scores was partly due to the different manipulations employed. The end results of this analysis were main effects that explained the influence of each manipulated independent variable on dependent variables (Field, 2013).

### 4.3. Results

Based on the results of Mauchly’s Test of Sphericity for employee value proposition, it was found that the assumption of sphericity was violated [χ^2^(5) = 20.312, *p* = 0.001]; thus, the reported results of the Greenhouse–Geisser corrected tests (*ε* = 0.937). The result of Within-Subjects Effects analysis showed that the employer value proposition at an e-grocery company significantly predicts millennials’ intention to work for that company, *F*(2.812, 584.856) = 15.515, *p* = 0.000. This then supported hypothesis 5a.

**Figure 6:**
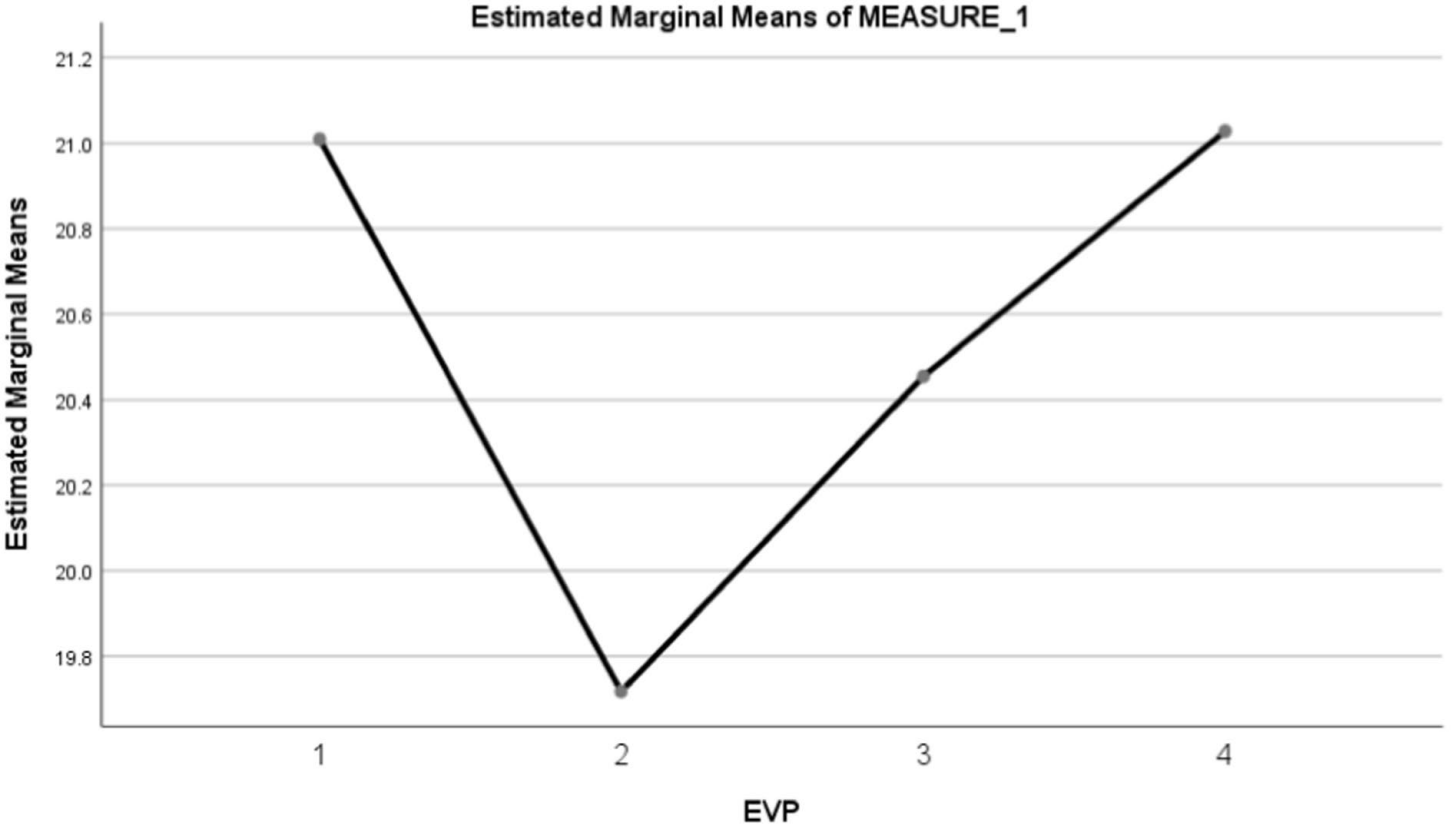
Descriptive statistics. Numbers on the horizontal axis indicate variations in employer/employee value propositions. The variations are (1) personal growth, (2) deeper connection, (3) radical flexibility, and (4) holistic well-being. All elements used in this image are created from Canva.com with subscription on 25 October 2021. All elements used in this image are created from Canva.com with subscription on 25 October 2021. It is copyrighted by Cindy Natalia Wijaya and may not reproduced without permission.

Based on Figure 6, the holistic well-being employee value proposition most predicts the intention of millennials in applying for a job at an e-grocery company (*M* = 21.03, SD = 2.940, SE = 0.203, 95% CI = [20.628, 21.430]). The holistic well-being value was not significantly different from the personal growth value (*M* = 21.01, SD = 3.084, SE = 0.213, 95% CI = [20.589, 21.430]). But these values were significantly different from radical flexibility value (*M* = 20.455, SE = 0.234, 95% CI = [19.994, 20.915]) and the value with the lowest influence was deeper connection (*M* = 19.72, SD = 3.889, SE = 0.269, 95% CI = [19.187, 20.248]). Thus, hypothesis 5b was not supported.

### 4.4. Discussion

The results suggest that post-pandemic employee value propositions promoted by Gartner (2021) significantly predict the intention of Indonesian millennials to apply for a job at the e-grocery company during the current pandemic. Many workers in Indonesia have experienced drastic changes in economic conditions and careers—such as job loss, decrease in wages, increase in workload due to a lack of employees, or concerns about working in an office prone to spreading COVID-19. This condition influenced the welfare of Indonesian millennial workers; thus, they required some benefits and protections from their workplaces to support their welfare, resilience, and families to survive (UNICEF et al., 2021). With the benefits and protections offered by companies, their intentions to apply increased, especially if the benefits were in line with their characteristics and needs. Conversely, according to the ILO Country Director Indonesia, Michiko Miyamoto, workers in Indonesia currently require family-friendly policies in their workplaces that support flexible work modalities. With flexible work modalities, workers can protect their families from COVID-19 risks while maintaining their jobs (UNICEF EAPRO et al., 2020). This finding brings a new explanation that the emergence of such new needs during the pandemic ultimately makes Indonesian workers interested in the benefits offered by post-pandemic employee value propositions. Millennials would be influenced to apply to those companies when e-grocery companies apply the post-pandemic employee value propositions.

Of the four types of post-pandemic employee value propositions available, holistic well-being was the value that attracted the most interest from Indonesian millennials. This is because mental health issues most often happen to people in the millennial generation (Winurini, 2020). They require higher mental health support from various parties, including the company they work at. Workers who work from home experience a special condition. They tend to spend more time with their families, thus making them have improved mental health and well-being. With improved mental health and well-being, they value well-being highly and hope that companies share a similar perspective (Izzatika et al., 2021). Lastly, according to the 2020 Behavioral Health Impact Update Research by The Standard (2020), millennials and gen-Zs are the generations that most often leave their jobs due to COVID-19 measures but also often forgo COVID-19 measures due to work demands. Thus, these two generations hope for mental health support in their workplaces. Consequently, these conditions give a new explanation that millennials would apply to an e-grocery company if they put holistic well-being forward as the main benefit when applying to an e-grocery company.

## 5. General discussion

As businesses that continue to grow after the COVID-19 pandemic, e-grocery companies must maintain the quality of their services to customers. One effort to improve quality is to recruit quality employees as workers who will provide services. In recruiting quality employees, e-grocery companies need a strategy that can make them compelling in the labor market and attract job seekers to apply. This strategy can be carried out through the selection of recruitment methods that will be followed, the establishment of employer branding as a promotional medium, and the provision of the most appropriate employee value proposition after the pandemic. This strategy must be able to attract millennials—the largest population in the labor market.

We conducted three experimental studies to examine the effect of these three strategies (e-recruitment method, employer branding, and post-pandemic employee value proposition) in predicting millennials’ intentions to apply to e-grocery companies. This study brings the novelty that only a few e-grocery companies use the e-recruitment method and have employer branding in Indonesia. The post-pandemic employee value proposition also has never been studied in Indonesia in the context of millennials in e-grocery companies. Using the experimental vignette method, more accurate and clear explanations of causality are provided related to these three strategies on millennials’ intentions to apply for jobs at e-grocery companies. The vignette used can elicit real millennial intentions in applying for jobs (Atzmüller and Steiner, 2010). This article may also give further knowledge for Indonesian e-grocery companies to win the talent war after this pandemic. They should pay attention to the simultaneous use of e-recruitment and employer branding, the content of their branding, and the post-pandemic benefit they should provide after this pandemic. It is important so that they can employ the best talents available that suit their business objective and organizational culture.

From the results of this research, it can be concluded that e-recruitment does not predict the intention of Indonesian millennials to apply for a job. This is not in line with Rimadias and Pratiwi (2017) research, although both were conducted in Indonesia but with different criteria. This is presumably due to differences in the scope of participants, where previous research only involved fresh graduates (with a maximum of 1-year work experience) from 3 universities in Jakarta. While for this study, participants consisted of all millennials in Indonesia with varying lengths of experience from 0 – over 5 years. The research method used is also different, where previous research only used descriptive research methods, while this study used the experimental vignette method with a certain stimulus given. In addition, in this study e-recruitment is displayed as a company website link which shows that recruitment will be carried out directly through the company’s official website. Whereas in previous research, e-recruitment was described in the form of commercial job boards. From this, it can be shown that e-recruitment using the website does not predict the intentions of millennial job seekers. Rimadias and Pratiwi (2017) also showed that millennial job seekers are more familiar with using certain job platforms such as JobStreet. The use of e-recruitment is also important in increasing effectiveness and company branding. But such applications must be socialized clearly to the candidate employees, especially when a company uses a platform such as the company’s official website. The use of e-recruitment and company websites as e-recruitment media also must be considered for socialization because it will help e-grocery companies to increase their attractiveness through the communication of their work culture and position themselves as the company of choice (Fountain, 2022).

The existence of employer branding can predict their intention. This is in line with many previous studies (Amelia and Nasution, 2016; Ruchika and Prasad, 2017; Purusottama and Ardianto, 2019; Kucherov and Zhiltsova, 2021). Employer branding builds trust for millennial job seekers who do not yet have a clear picture of the e-grocery company they are applying for. Like a customer in the labor market, these millennial job seekers will be more interested in companies that can provide a complete picture so that they can adjust what the company needs with their values, experience, and characteristics (Putritamara et al., 2022). According to the Theory of Planned Behavior (Ajzen, 1985), when the evaluation and attitude toward a company are positive and in accordance with the conditions of the job seeker, their intention to apply will be even greater.

The influence of employer branding will be more beneficial because of the collectivist culture in Indonesia. Indonesian people (including millennials) tend to prioritize relationships and togetherness (Cultural Atlas Editors, 2016). They tend to be often driven to do something because of the relationships and trust formed and the sense of togetherness that exists when doing things considered positive by most individuals. Through employer branding, companies can build relationships and trust with the Indonesian people, encouraging them to apply for jobs. Moreover, with candid reviews from employees and benefits personalized to the times’ needs, employer branding can touch job seekers personally and make them feel that they have the same needs and values, which ultimately fosters their sense of community and encourages them to apply to the company, like other employees who have joined. Indonesian people will also generally see themselves as doing business with people, not entities. The employer branding will represent the company as an individual with the same value and understanding of the jobseeker’s need, more than what the company does.

Then, the simultaneous application of e-recruitment and employer branding on an e-grocery company could encourage a greater intention of millennials to apply to e-grocery companies. The implementation of e-recruitment will serve as a means of branding in promoting the system and work culture of the company. Employer branding can be a means of socializing to prospective candidates regarding how to carry out online recruitment, which may not be too familiar to them (Sabha, 2018). With this further socialization, the effectiveness and ease of use of e-recruitment will be conveyed and encourage millennial job seekers to use it.

From Study 1, e-grocery companies should promote company websites through an attractive employer branding activity as an effective recruitment method. E-grocery companies could also maximize employer branding as a medium to promote the company’s vision, mission, and values, which would increase candidate employees’ interest and trust in the company. The use of e-recruitment and employer branding should bring together the e-grocery company, as the interaction of these variables could bring greater intention to the millennials.

Our results also concluded that employer branding could still predict millennials’ intention to apply for a job at the e-grocery company when the length of work experience is controlled. That is, the length of work experience of millennial job seekers does not determine how they perceive employer branding itself. However, as a “customer” in the labor market (Gallup, 2016), our results also concluded that employer branding could still predict millennials’ intention to apply for a job at the e-grocery company when the length of work experience is controlled. That is, the length of work experience of millennial job seekers does not determine how they perceive employer branding itself. However, as a “customer” in the labor market (Buzzeo and Cifci, 2017). The quality of candidates’ experience would influence their perception of the company. Thus, we can suggest that e-grocery companies should consider the quality of the candidate’s experience, instead of their length, when developing the employer branding content/activity. As employer branding still influences them in applying for jobs, providing positive recruitment and marketing experience would be good, so they can build their intentions to apply at e-grocery companies.

For the last study, it can be concluded that the post-pandemic employee value proposition in employer branding can predict the intention of millennials. Due to changing conditions brought about by the pandemic, job seekers now have different needs and expectations when applying for jobs. By prioritizing a more human–deal value proposition and benefits, job seekers have 15% higher job satisfaction than before (Gartner, 2021). Of the four existing post-pandemic employee value propositions, the holistic well-being type was the most attractive post-pandemic employee value proposition, especially because of the condition of workers who experienced anxiety, fear, and uncertainty during the COVID-19 pandemic, impacting their welfare (UNICEF et al., 2021). With the employee value proposition holistic well-being, employees are not only provided facilities to support mental well-being but are also supported physically and spiritually, meaning that the company supports the promotion of employee welfare at work (Gartner, 2021). Applying the post-pandemic employee value proposition is also beneficial in Indonesia because of the culture of harmony in Indonesian society (Cultural Atlas Editors, 2016; Emerhub, 2017). The culture of harmony puts forward the existence of self-harmony when working. Not only being urgently productive but also maintaining a fun/pleasant atmosphere when working. The existence of good harmony is the most important component to increasing business. Therefore, applying this type of holistic well-being is one of the main attractions for jobseekers because of the encouragement to align themselves in the company when they join, according to the culture of harmony in society.

From this conclusion, we suggest that e-grocery companies should try to bring the post-pandemic employee value propositions to their companies in adapting to the post-pandemic condition. This adaptation process can be started by providing additional benefits to employees, to the internalization of work values, all of which are included in the post-pandemic employee value proposition. From the available post-pandemic employee value propositions, e-grocery companies can prioritize providing welfare facilities in the workplace and encourage workers to use that facility. The facilities may include free counseling for employees, sports and health facilities, manager–employee sharing, and so on. Apart from attracting new employees to join the company, the adopted employee value propositions will help companies keep the present employees as companies try to fulfill their current needs.

This research has some limitations. For instance, (1) this research only obtained a minimum number of subjects in each of its studies (only 200 subjects per study), thus it is not representative of the entire population of millennials in Indonesia; (2) the vignette used only focused on describing the recruitment conditions in an e-grocery company, and so it cannot be generalized to the extent of the conditions of companies from other sectors; and (3) this research only took samples from several areas in Indonesia, thus it cannot be generalized to other countries.

For future research, we suggest that the number of research samples should be increased, and there should be samples from various areas in Indonesia to show the condition of Indonesian millennials accurately. Then, future research on a similar topic can be carried out on companies in sectors other than e-grocery. Future research should also consider the impact of cultural background in developing branding. Lastly, research may be carried out to test the influence of the recruitment process on the intention of jobseekers to join a company.

## Data availability statement

The raw data supporting the conclusions of this article will be made available by the authors, without undue reservation.

## Ethics statement

The studies involving human participants were reviewed and approved by the Ethical Clearance from the Committee of Ethics, Faculty of Psychology, Universitas Indonesia (No. 155/FPsi.Komite Etik/PDP.04.00/2021). The patients/participants provided their written informed consent to participate in this study.

## Author contributions

CW designed and planned the experiment, collected and analyzed the data, and wrote the manuscript. MM refined the experiment instruments and tools, interpreted the data, and refined the manuscript. SB and BB provided critical evaluations of the manuscript. All authors contributed to the article and approved the submitted version.

## Funding

This research was financially supported by the Universitas Indonesia through the 2022 Universitas Indonesia Research Grant program, the International Indexed Publication (PUTI) scheme Q2 batch 3 (No. 2116/SK/R/UI/2022).

## Conflict of interest

The authors declare that the research was conducted in the absence of any commercial or financial relationships that could be construed as a potential conflict of interest.

## Publisher’s note

All claims expressed in this article are solely those of the authors and do not necessarily represent those of their affiliated organizations, or those of the publisher, the editors and the reviewers. Any product that may be evaluated in this article, or claim that may be made by its manufacturer, is not guaranteed or endorsed by the publisher.
